# Potential link between high FIB-4 score and chronic kidney disease in metabolically healthy men

**DOI:** 10.1038/s41598-022-21039-0

**Published:** 2022-10-05

**Authors:** Akihiro Kuma, Kosuke Mafune, Bungo Uchino, Yoko Ochiai, Tetsu Miyamoto, Akihiko Kato

**Affiliations:** 1 Kidney Center, Hospital of the University of Occupational and Environmental Health, 1-1 Iseigaoka, Yahatanishi-ku, Kitakyushu, Fukuoka 807-8556 Japan; 2 Blood Purification Unit, Hamamatsu University Hospital, 1-20-1 Handayama, Higashi-ku, Hamamatsu, Shizuoka 431-3192 Japan; 3 Department of Mental Health, Institution of Industrial Ecological Sciences, University of Occupational and Environmental Health, 1-1 Iseigaoka, Yahatanishi-ku, Kitakyushu, Fukuoka 807-8555 Japan; 4 Health Promotion Center, Yamaha Motor Co. Ltd., 2500 Shingai, Iwata, Shizuoka 438-8501 Japan

**Keywords:** Occupational health, Nephrology

## Abstract

Although the association between non-alcoholic fatty liver disease and chronic kidney disease (CKD) has been well known, it is unclear whether Fibrosis-4 (FIB-4) score is a predictor of CKD development. We performed this retrospective cohort study, with a longitudinal analysis of 5-year follow-up data from Japanese annual health check-ups. Participants with CKD (estimated glomerular filtration rate [eGFR] < 60 mL/min/1.73 m^2^ and/or proteinuria) and a habit of alcohol consumption were excluded. The cut-off FIB-4 score was 1.30, indicating increased risk of liver fibrosis. Overall, 5353 participants (men only) were analyzed without exclusion criteria. After propensity score matching, high FIB-4 score (≥ 1.30) was not an independent risk factor for incident CKD (odds ratio [OR] 1.57; 95% confidence interval [CI] 0.97–2.56). However, high FIB-4 score was a significant risk factor for CKD in non-obese (OR 1.92; 95% CI 1.09–3.40), non-hypertensive (OR 2.15; 95% CI 1.16–3.95), or non-smoking (OR 1.88; 95% CI 1.09–3.23) participants. In these participants, FIB-4 score was strongly associated with eGFR decline in the multiple linear regression analysis (*β* = − 2.8950*, P* = 0.011). Therefore, a high FIB-4 score may be significantly associated with CKD incidence after 5 years in metabolically healthy participants.

## Introduction

Chronic liver disease causes mortality, morbidity, and health care resource utilization^1^. The prevalence of non-alcoholic fatty liver disease (NAFLD), a chronic liver disease, is increasing proportionately with epidemics such as obesity, hypertension, and type 2 diabetes mellitus^2,3^. In fact, the prevalence of NAFLD in the general population is approximately 25% worldwide^4^. Some patients with NAFLD have a high risk of advanced liver fibrosis, cirrhosis, portal hypertension, hepatocellular carcinoma, or death.

Both NAFLD and chronic kidney disease (CKD) progress to chronic conditions that present a spectrum of mild to severe diseases with end-stage damage. The known risk factors for NAFLD are similar to those of CKD: obesity, hypertension, diabetes mellitus, or dyslipidemia^5–10^. Previous reports show that NAFLD is a risk factor for the incidence of CKD^11,12^. Patients with NAFLD had higher abnormal urine albuminuria than those without NAFLD^13^. Furthermore, NAFLD was associated with early microalbuminuria (≥ 30 mg/gram creatinine) and decreased estimated glomerular filtration rate (eGFR)^12^. Recently, several cross-sectional studies have indicated that the prevalence of stage 3 CKD (defined as < 60 mL/min/1.73 m^2^ of eGFR) was markedly increased in patients with NAFLD under adjusted multiple co-variances^14^. However, the association between NAFLD and eGFR level below 60 mL/min/1.73 m^2^ remains unknown.

NAFLD is a significant feature of hepatic fibrosis and inflammation; therefore, liver biopsy is required for diagnosis. However, liver biopsy is an invasive test and may result in sampling error due to the small sample size^15,16^. The Fibrosis-4 (FIB-4) score, one of the several clinical scoring systems for liver fibrosis, has been developed as a noninvasive panel to stage liver fibrosis calculated by age and blood test results. The FIB-4 score had the best diagnostic accuracy for advanced liver fibrosis analyzed by receiver operating curves (area under the curve, 0.86) compared to other scoring systems^17,18^. A FIB-4 score < 1.30 had a > 90% negative predictive value for fibrosis^17,18^. Xu et al. showed that FIB-4 scores ≥ 1.10 were significantly associated with the prevalence of CKD in patients with NAFLD^19^; however, the study was cross-sectional and targeted to patients with NAFLD. Our study was a longitudinal 5-year follow-up study which aimed to determine the association between CKD incidence and FIB-4 score in the men.

Most previous studies investigating the association between liver disease or liver fibrosis—including NAFLD—and kidney disease were cross-sectional. Furthermore, their subjects were not the general population. As described above, the FIB-4 score is useful as a predictor of liver fibrosis, but the relationship between incident CKD and FIB-4 score has not been well-investigated. Therefore, we aimed to investigate the association between FIB-4 score and incident CKD in a longitudinal 5-year follow-up study.

## Results

### Participant characteristics and propensity score matching

**Figure 1:**
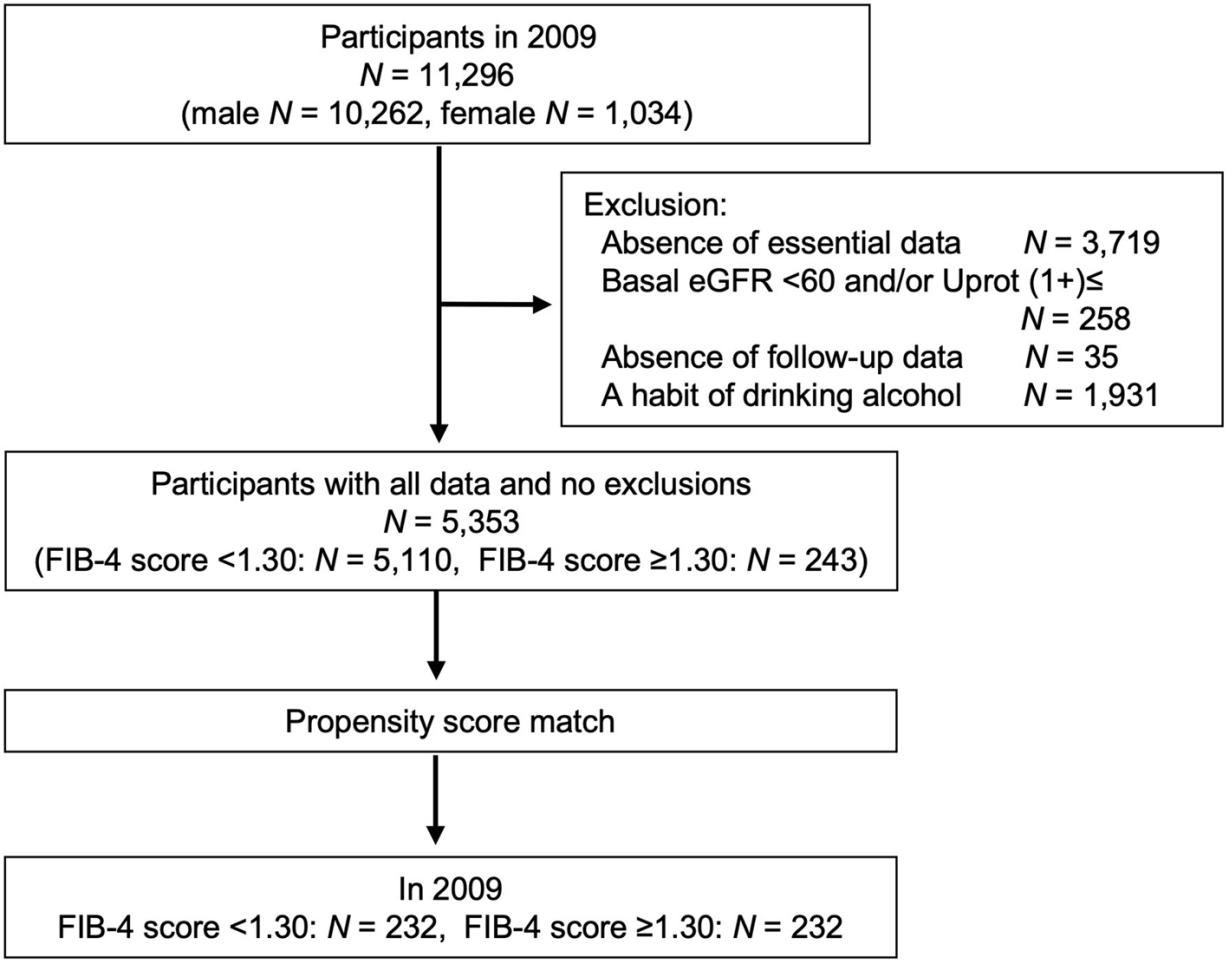
Enrollment, exclusion, and propensity score matching. The data collected included age, body mass index, estimated glomerular filtration rate (eGFR, mL/min per 1.73 m^2^), transaminase, platelet, lipid profile, hemoglobin A1c, serum uric acid, blood pressure, urinalysis, and self-interview sheet. Participants with missing essential data from the 5-year follow-up were excluded. The habit of drinking alcohol was defined as consumption of ≥ 30 g/day (men) and ≥ 20 g/day (women) of ethanol. In the exclusion criteria process, all women (*N* = 1034) were left out, so analyzed participants (*N* = 5353) were men only. Uprot; proteinuria with dipstick testing.

At baseline there were 10,262 men and 1034 women; however, 5943 participants were excluded according to the exclusion criteria, and all women were excluded in the exclusion criteria process (Fig. 1). The enrolled participants (*N* = 5353) were divided into two groups according to a basal FIB-4 score of < 1.30 (low risk of liver fibrosis) or ≥ 1.30 (moderate to high risk of liver fibrosis). In this cohort, the mean FIB-4 score and eGFR were 0.73 and 81.8 mL/min/1.73 m^2^, respectively (Supplementary Table S1). The proportion of participants with a high FIB-4 score (≥ 1.30) was 4.5% (*N* = 243). Before adjusting for co-variants, basal age, eGFR, systolic blood pressure, hemoglobin A1c, and frequency of daily smoking habits were significantly different between the two groups. After propensity score matching, the comparison of participant backgrounds between the two groups showed no significant differences in all clinical parameters—except AST, ALT, and platelets—that were used to calculate the FIB-4 score (Table 1). The standardized mean differences also showed no significant differences between the two groups (Supplementary Fig. S1).

**Table 1:** Baseline characteristics of participants.

	Unadjusted				After propensity score matching				
	FIB-4 < 1.30	FIB-4 ≥ 1.30	p value	Standardized differences	FIB-4 < 1.30	FIB-4 ≥ 1.30		p value	Standardized differences
Participants, n	5110	243			232	232			
Age, years	37 (10)	51 (7)	< 0.0001	1.615	50 (7)	50 (7)		0.8477	0.014
FIB-4 score	0.69 (0.23)	1.65 (0.48)	< 0.0001		0.91 (0.22)	1.65 (0.49)		< 0.0001	
Body mass index, kg/m^2^	22.9 (3.3)	22.9 (3.6)	0.8498	− 0.012	22.9 (2.7)	22.9 (3.6)		0.8244	0.019
eGFR, mL/min/1.73 m^2^	82.1 (12.4)	73.9 (10.6)	< 0.0001	− 0.717	74.7 (9.9)	73.9 (10.7)		0.4408	− 0.064
AST, IU/L	23 (9)	35 (36)	< 0.0001		23 (7)	35 (37)		< 0.0001	
ALT, IU/L	27 (21)	31 (26)	0.0266		25 (15)	31 (26)		0.0046	
γ-GTP, IU/L	34 (30)	42 (46)	0.0001		38 (35)	41 (41)		0.465	
Triglycerides, mg/dL	109 (77)	99 (86)	0.0451	− 0.125	98 (53)	100 (88)		0.7573	0.026
LDL-cholesterol, mg/dL	119 (32)	119 (30)	0.9077	− 0.008	117 (26)	120 (30)		0.7322	0.029
Platelet, × 10^3^/μL	257 (49)	191 (41)	< 0.0001		262 (51)	191 (42)		< 0.0001	
Uric acid, mg/dL	6.0 (1.1)	5.9 (1.2)	0.4556	− 0.048	5.9 (1.1)	5.9 (1.2)		0.9624	− 0.004
Hemoglobin A1c, %	4.8 (0.5)	5.0 (0.7)	< 0.0001	0.316	4.9 (0.5)	5.0 (0.7)		0.3167	0.093
Systolic blood pressure, mmHg	118 (13)	121 (18)	0.0002	0.215	122 (17)	122 (18)		0.987	0.002
Smoking, n (%)	1908 (37)	56 (23)	< 0.0001	0.315	50 (22)	56 (24)		0.507	− 0.057
**Medication**									
Hypertension, n (%)	143 (3)	18 (7)	< 0.0001		25 (11)	17 (7)		0.1955	
Diabetes mellitus, n (%)	82 (2)	11 (5)	0.0007		7 (3)	11 (5)		0.4718	

Propensity score matching (1:1) was performed by covariates such as age, body mass index, eGFR, triglycerides, LDL-cholesterol, hemoglobin A1c, uric acid, systolic blood pressure, and smoking habit. Smoking: participants with daily habit of smoking. Data are expressed as mean (standard deviation), except smoking and medication for hypertension and diabetes mellitus.

*ALT* alanine transaminase, *AST* aspartate transaminase, *eGFR* estimated glomerular filtration rate, *γ-GTP* gamma-glutamyl transferase, *LDL* low-density lipoprotein.

*P* value was calculated by Student’s t-test or χ^2^ test.

### High-level of FIB-4 score was associated with incident chronic kidney disease

**Figure 2:**
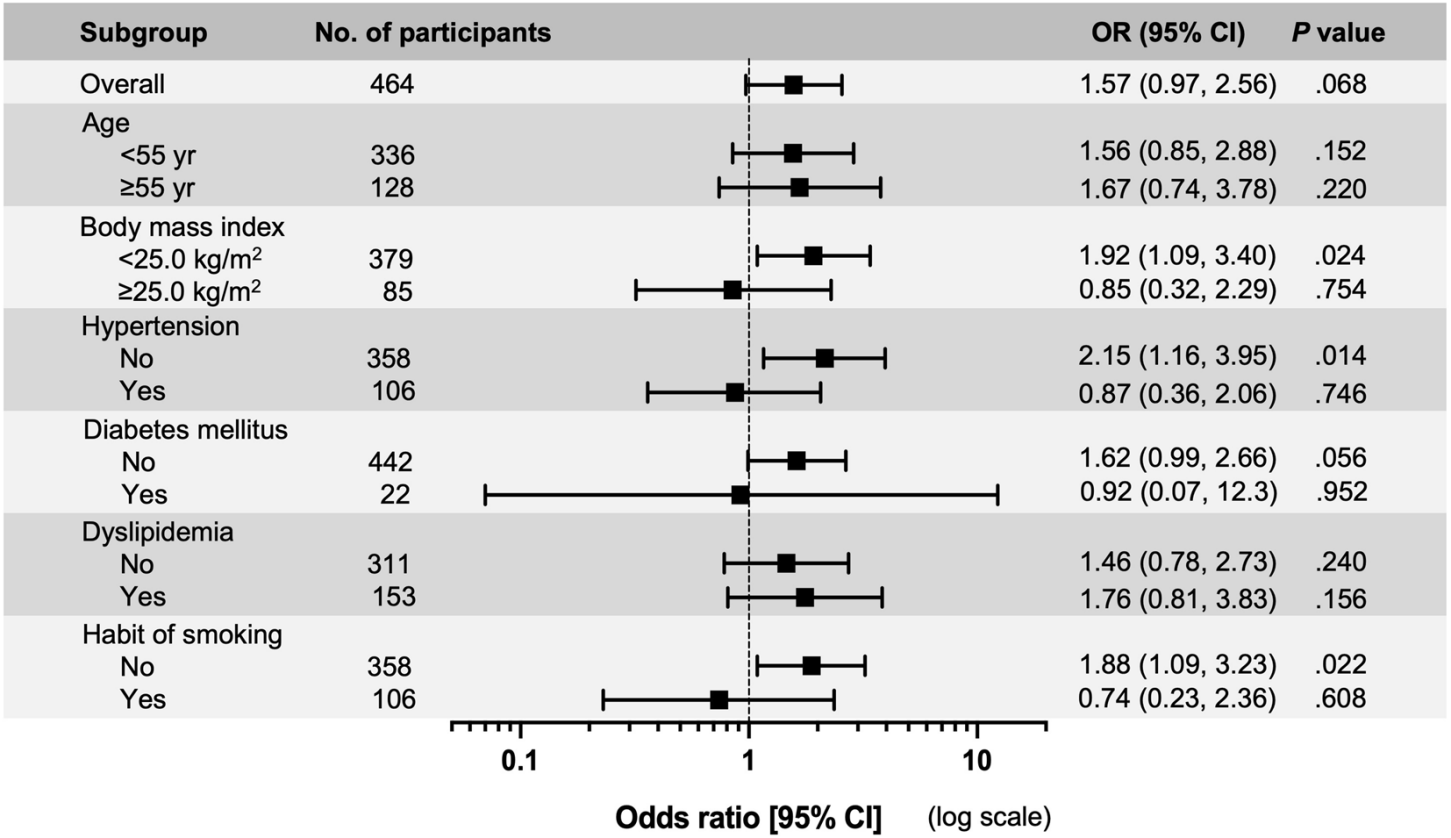
Risk of high-level FIB-4 score for incident chronic kidney disease after a 5-year follow-up. Odds ratios (OR) were calculated after propensity score matching. Hypertension was defined as systolic blood pressure ≥ 140 mmHg, diastolic blood pressure ≥ 90 mmHg, and/or the use of antihypertensive medication. Diabetes mellitus was defined as a hemoglobin A1c level ≥ 6.5% and/or the use of anti-diabetes mellitus medicine, including insulin. Dyslipidemia was defined as a triglyceride level ≥ 150 mg/dL and/or low-density lipoprotein cholesterol level ≥ 140 mg/dL.

We analyzed the risk of incident CKD after a 5-year follow-up according to the FIB-4 score (Fig. 2). Overall, a high FIB-4 score (≥ 1.30) was not a significant risk factor for the development of CKD (odds ratio (OR) 1.57; 95% confidence interval (CI) 0.97–2.56). The amount of incident CKD and newly developed CKD was 33 (14%) in participants with low-level FIB-4 scores and 48 (21%) in participants with high-level FIB-4 scores. Next, subgroups were formed into two groups according to age, body mass index (BMI), hypertension, diabetes mellitus, dyslipidemia, or smoking habits. A high FIB-4 score was significantly associated with incident CKD (OR 1.92; 95% CI 1.09–3.40) in participants without obesity, but not in those with obesity. Furthermore, a high FIB-4 score was also significantly associated with incident CKD in participants without hypertension (OR 2.15; 95% CI 1.16–3.95) or smoking (OR 1.88; 95% CI 1.09–3.23). However, age, diabetes mellitus, and dyslipidemia did not affect CKD development.

### In metabolic healthy participants, FIB-4 score was strongly associated with CKD development

**Figure 3:**
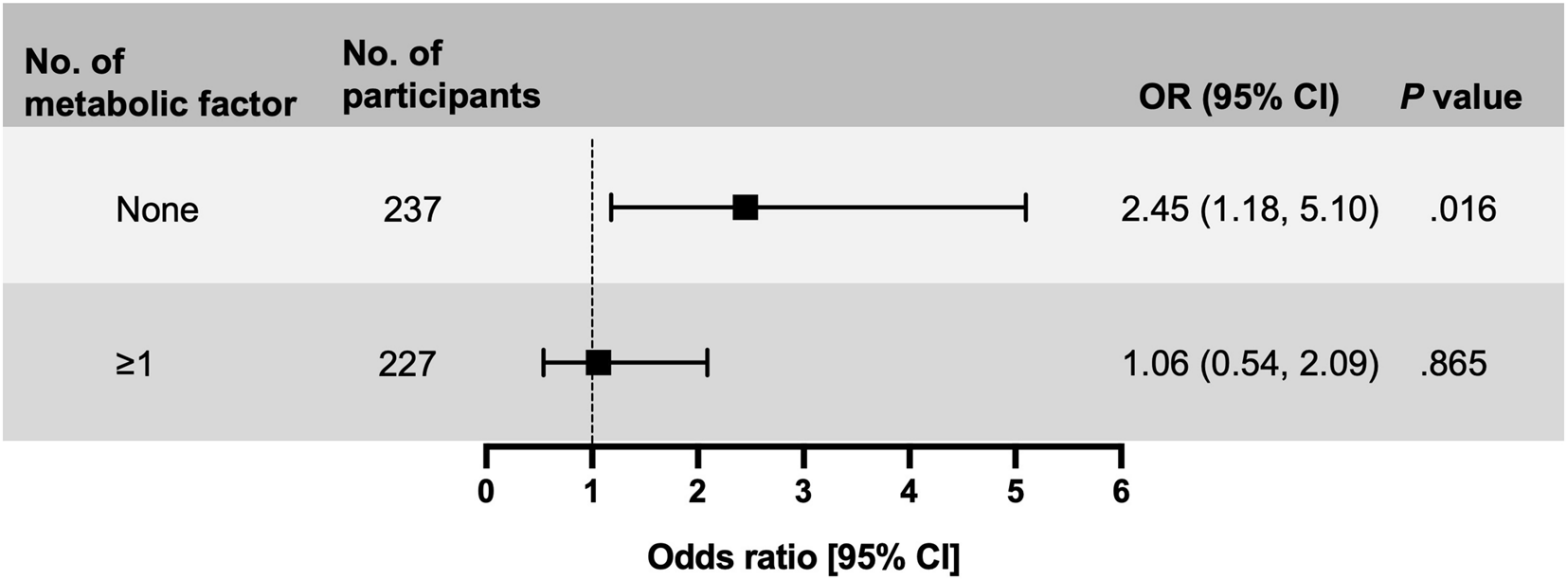
High-level FIB-4 score was a risk factor for incident chronic kidney disease after a 5-year follow-up, by the number of metabolic factors. Odds ratios (ORs) were calculated after propensity score matching. Metabolic factors included body mass index ≥ 25.0 kg/m^2^, hypertension (defined as systolic blood pressure ≥ 140 mmHg, diastolic blood pressure ≥ 90 mmHg, and/or the use of antihypertensive medication), or daily smoking habits.

We showed three metabolic statuses related to incident CKD: BMI, hypertension, and smoking habits (Fig. 2). Participants (*N* = 464) were divided into two groups according to the status of the metabolic factors, and the risk of CKD development was investigated using the FIB-4 score (Fig. 3). In participants who had no metabolic factors (*N* = 237), a high FIB-4 score was significantly associated with CKD incidence (OR 2.45; 95% CI 1.18–5.10). Next, we performed a sensitivity analysis on participants without the three metabolic factors using linear regression analysis (Table 2, Supplementary Fig. S2). The FIB-4 score was strongly associated with the rate of change in eGFR. However, if the participants had at least one of the three metabolic factors, a high FIB-4 score was not a risk factor for incident CKD, and the FIB-4 score was not related to the rate of change in eGFR (Fig. 3, Supplementary Table S2).

**Table 2:** Linear regression analysis with the rate of change in eGFR as dependent variables in participants without specific metabolic abnormal.

Variables	Simple linear regression				Multiple linear regression				
	Coefficient	95% CI	t-value	p value	Standardized coefficient	Coefficient	95% CI	t-value	p value
FIB-4 score	− 2.9146	− 5.1696 to − 0.6596	− 2.55	0.012	− 0.1628	− 2.895	− 5.1133 to − 0.6767	− 2.57	0.011
Age	− 0.0976	− 0.2615 to 0.0662	− 1.17	0.242					
Body mass index	0.1119	− 0.5134 to 0.7371	0.35	0.725					
Triglycerides	− 0.0161	− 0.0304 to − 0.0019	− 2.23	0.027	− 0.1419	− 0.0159	− 0.0299 to − 0.0019	− 2.24	0.026
LDL-cholesterol	0.0213	− 0.0227 to 0.0652	0.95	0.341					
Uric acid	1.1348	0.1291 to 2.1404	2.22	0.027	0.1371	1.0838	0.0974 to 2.0702	2.16	0.031
Hemoglobin A1c	0.7426	− 2.3511 to 3.8364	0.47	0.637					
Systolic blood pressure	− 0.0156	− 0.1122 to 0.0810	− 0.32	0.751					

N = 237; Model adjusted R^2^, 0.055; Model F, 5.55; P = 0.0011; Rate of change in eGFR = (eGFR_2014_ − eGFR_2009_)/eGFR_2009_.

Metabolic factors included body mass index ≥ 25.0 kg/m^2^, hypertension (defined as systolic blood pressure ≥ 140 mmHg, diastolic blood pressure ≥ 90 mmHg, and/or the use of antihypertensive medication), or daily smoking habits.

*eGFR* estimated glomerular filtration rate, *LDL* low-density lipoprotein.

## Discussion

In this retrospective and longitudinal study, we analyzed health check-up data of over 10,000 Japanese participants, and the propensity score matching picked approximately 500 men for the analysis. The major finding of our study was that a high FIB-4 score (≥ 1.30) was significantly associated with the development of CKD in participants without obesity, hypertension, and/or habit of daily smoking, although the FIB-4 score was not significantly associated with the overall incidence of CKD. When participants had none of these three metabolic factors, the FIB-4 score may strongly predict eGFR decline from the sensitivity analysis through linear regression (Table 2). These findings may contribute to the prevention of kidney dysfunction in men with NAFLD, even though they appear metabolically healthy.

In this study, we used the FIB-4 score to assess liver damage as an indicator of NAFLD. To identify liver fibrosis, AST/ALT ratio, FIB-4 score, NAFLD fibrosis score, and BARD score were used; the FIB-4 score had the best diagnostic accuracy compared to other scoring systems^17^. A FIB-4 score of 1.30 was associated with an eGFR < 60 mL/min/1.73 m^2^ 5 years later in participants who appeared metabolically healthy (Fig. 3). A low cut-off point (< 1.30) of the FIB-4 score had a 90% negative predictive value for advanced liver fibrosis stage 3–4, which implied high mortality and hepatic cirrhosis^18^.

A high FIB-4 score (≥ 1.10 or ≥ 1.30) was significantly associated with the prevalence of CKD in patients with NAFLD diagnosed by liver biopsy or ultrasound, but not CKD incidence^19,20^. In retrospective cohort studies in the general population, a higher FIB-4 score was also associated with the prevalence of CKD and eGFR decline, but not CKD incidence^21,22^. Furthermore, a FIB-4 score ≥ 1.30 was a significant risk for eGFR < 60 mL/min/1.73m^2^ and/or proteinuria in patients with type 2 diabetes mellitus^23^. As described above, in our cohort, a FIB-4 score ≥ 1.30 was also not associated with CKD incidence over a 5-year follow-up. However, in our study, a FIB-4 score ≥ 1.30 became a risk factor for CKD incidence in participants without hypertension, obesity, or a current smoking habit (low-risk group of CKD). This result may be beneficial for occupational and public health to prevent CKD incidence.

Liver fibrosis and kidney dysfunction are associated with each other and develop together. Previous studies showed that metabolic disorders, such as hyperglycemia, obesity, or dyslipidemia, induced both liver fibrosis and kidney dysfunction^21,24,25^. Thus, there was not clear for the causal relation between liver fibrosis and kidney dysfunction. In contrast, our study showed that higher FIB-4 scores (≥ 1.30) and liver fibrosis may become a risk for CKD incidence with adjusted co-variables of several metabolic parameters, such as BMI, HbA1c, LDL-C, and TG, by propensity score matching.

Liver fibrosis development activates the renin-angiotensin system (RAS) and synthesizes angiotensin II^26,27^. Subsequently, the activated RAS induces kidney fibrosis and kidney dysfunction through remodeling and systematic hypertension. Furthermore, liver fibrosis produces chronic inflammation and oxidative stress by peroxisome proliferator-activated receptor α^28^, which can lead to kidney injury. As well as, in the status of NAFLD. Experimental evidence indicates that interstitial microbiota and platelet activation play a role in the pathogenesis of CKD or NAFLD through oxidative stress, uremic toxin, and inflammation^29–32^.

The National Health and Nutrition Examination Survey (NHANES)-III database support that metabolic dysfunction-associated fatty liver disease (MAFLD) is more strongly associated with CKD prevalence than with NAFLD. Patients with MAFLD had multiple metabolic comorbidities (e.g., hypertension, type 2 diabetes mellitus, and obesity) and higher Homeostatic Model Assessment for Insulin Resistance^33^. Interestingly, our study showed that the FIB-4 score was strongly associated with incident CKD when participants were non-obese, non-hypertensive, and non-smokers (Fig. 2). These findings indicate that NAFLD and liver fibrosis may be independent risk factors for incident CKD.

There was a reverse relationship between the risk of CKD incidence and the number of metabolic factors (Supplementary Table S3). Obesity, hypertension, and smoking habit, which are well-known as important and strong risk factors of kidney dysfunction, may weaken the prediction of FIB-4 score for CKD incidence. In contrast, our study found that the FIB-4 score can become a useful predictor for CKD incidence in the general population (metabolically healthy), although they have ever seemed to be low-risk group for CKD.

NAFLD is associated with obesity. However, the prevalence of lean NAFLD (NAFLD with BMI < 25.0 kg/m^2^) was 5–26% in the Asian population and 7–20% in the Western population^34^. In the NHANES-III database, lean NAFLD was independently associated with younger age, female sex, insulin resistance, and dyslipidemia. In addition, lean NAFLD is associated with lower levels of proinflammatory cytokines (e.g., interleukin 6 and tumor necrosis factor alpha), which are known as a risk factors for cardiovascular disease, metabolic syndrome, and insulin resistance. Furthermore, in Japanese NAFLD patients, the FIB-4 score in lean NAFLD patients was higher than that in NAFLD patients with obesity, and fatty liver was not milder^35^. Therefore, although non-obese people are often ignored as a high-risk group for NAFLD and CKD, it is important to identify lean NAFLD; this may contribute to reducing the frequency of CKD patients.

Atherogenic dyslipidemia, which is characterized by hyperglycemia, decreased high-density lipoprotein, and increased low-density lipoprotein cholesterol, is often observed in patients with NAFLD. Dyslipidemia linked to hepatic lipid metabolism and hepatic steatosis promotes cardiovascular disease^36,37^. In addition, dyslipidemia is known to be a risk factor for kidney dysfunction^6,8,38,39^. In this study, a high FIB-4 score was not a significant risk factor for the development of CKD, regardless of lipid profile status. We believe that 42% of participants without dyslipidemia had at least one of the three metabolic factors (obesity, hypertension, or smoking habit); therefore, the FIB-4 score was not strongly associated with kidney function.

There were some limitations in this study. First, it did not include women. The original cohort included approximately 9% female participants; however, all women were finally excluded in the process of the exclusion criteria. Therefore, our findings may not be relevant to women. Second, the self-interview sheet did not include information on comorbidities and medication, except for antihypertensive and anti-diabetes mellitus medication use. Therefore, covariates such as comorbidities and medication were excluded from the statistical analysis. Third, FIB-4 score estimates the amount of scarring in the liver. Some liver diseases, such as hepatitis B, hepatitis C, autoimmune liver disease, or drug-induced liver disease, contribute to liver fibrosis. However, this study did not exclude participants with those liver diseases, because annual health check-ups did not perform blood tests for liver diseases and liver biopsy. In addition, the questionnaire (self-interview sheet) did not include the treatment and history of liver diseases. Finally, we analyzed only eGFR and proteinuria during health check-ups, although the Kidney Disease Improving Global Outcome guidelines suggest that multiple tests are necessary to diagnose CKD. Our patients only underwent blood and urine tests during an annual health check-up; however, we could not request patients to undergo blood and urine tests several times a year due to the retrospective nature of the study.

In summary, a high FIB-4 score (≥ 1.30) was significantly associated with incident CKD 5 years later in healthy men, such as those with no obesity, hypertension, or smoking habits. Therefore, metabolically healthy NAFLD patients may be at risk for incident CKD. Our findings suggest that patients with NAFLD and FIB-4 score ≥ 1.30 who are metabolically healthy should be carefully observed for kidney function, although they appear to have a low risk of developing CKD.

## Methods

### Study design, setting, and population

This retrospective, observational study involved the health check-up data and self-interview sheets of laborers supplied from an enterprise in Japan. Blood and urine test results from health check-ups conducted from 2009 to 2014 were collected and analyzed. The main outcome in this study was the development of CKD over a 5-year follow-up depending on the basal FIB-4 score. Incident CKD was defined as < 60 mL/min/1.73 m^2^ of eGFR and/or proteinuria (≥ 1+) with the dipstick method. In 2009, 11,296 participants (10,262 men and 1034 women) were enrolled. Participants were excluded according to the following criteria: basal eGFR < 60 mL/min/1.73 m^2^ and/or proteinuria with the dipstick test (*N* = 258) and missing 5-year follow-up data (*N* = 35) (Fig. 1). Furthermore, workers with a habit of alcohol consumption (*N* = 1931) were excluded because the target of this study was NAFLD or non-alcoholic steatohepatitis as a cause of incident CKD. A habit of alcohol consumption was defined as ≥ 30 g/day (men) and ≥ 20 g/day (women) of ethanol, according to the guidelines of the Japanese Society of Gastroenterology^40^. In the exclusion process, all women were excluded. In this study, participants were divided into two groups based on basal FIB-4 score (low risk of liver fibrosis, FIB-4 score < 1.30; moderate and high risk of liver fibrosis, FIB-4 score ≥ 1.30)^17^. When the cut-off value of FIB-4 score was 1.30, the positive predictive and negative predictive values were 36% and 95%, respectively, for advanced liver fibrosis in patients with NAFLD^17^. Participant baseline characteristics (*N* = 5353) are shown in Supplementary Table S1.

### Ethics approval

The study was conducted following the 1964 Helsinki Declaration and its later amendments or comparable ethical standards, and was approved by the Hamamatsu University School of Medicine ethics committee/institutional review board (approval No. E15-289). The study was exempted from informed consent requirements owing to its retrospective design. All participants were given the means to opt-out of the study. The opt-out notification was displayed in the clinic where the participants underwent health check-ups and on the home page of Hamamatsu University Hospital. All methods complied with relevant guidelines and regulations.

### Data sources/health check-up data

The participants’ body height, weight, and blood pressure (BP) were measured at each health check-up. BMI was calculated as body weight (kg) divided by height squared (m^2^). eGFR was calculated using the following equation: eGFR (mL/min/1.73 m^2^) = 194 × serum creatinine^−1.094^ × age^−0.287^, as described previously^41^. The change in eGFR during the 5-year follow-up was assessed using the following equation: rate of change in eGFR = (eGFR_2014_ − eGFR_2009_)/eGFR_2009_. The FIB-4 score was calculated using the following equation: FIB-4 score = age × AST (IU/L)/platelet count (× 10^9^/L) × √ALT (IU/L)^42^. Blood and urine samples were analyzed in a private laboratory. The self-interview sheet included information on the participants’ habit of current smoking and alcohol consumption. Smoking habit was defined as the current daily smoking status. For the analysis, we excluded participants with a habit of drinking alcohol.

### Statistical analysis

To describe the non-equivalence of our cohort, we performed propensity score matching with logistic regression (SAS version 9.2, SAS Institute Inc., NC, USA)^43,44^. The cohort was formed based on 1:1 nearest-neighbor matching using a greedy algorithm. For the propensity score model, we adjusted the following co-variances: basal eGFR, age, BMI, low-density lipoprotein cholesterol, triglycerides, hemoglobin A1c, systolic BP, serum uric acid, and smoking habit.

This process matched 232 participants (95.5% of high-level FIB-4 score participants) with the same number of control participants (Table 1). A normal distribution of all clinical parameters was confirmed by the Kolmogorov–Smirnov test. A statistical difference in background characteristics between the two groups (matched high-level FIB-4 score participants and control participants) was analyzed using a two-tailed Student’s t-test or chi-square test and standardized mean differences (Supplementary Fig. S1). OR for CKD development were determined using logistic regression. Additionally, a linear regression analysis for the rate of change in eGFR was performed as a sensitivity analysis. Statistical significance was set at p < 0.05. The Student’s t-test, chi-square test, logistic regression test, and linear regression were performed using Stata 12SE (Stata Co., College Station, TX, USA).

## Supplementary Information

Supplementary Information.

## Data Availability

The datasets analyzed during the current study are restricted for use for security reasons as these were obtained from third parties. However, they are available from the corresponding author on reasonable request.
