# Supplementary material for: Potential link between high FIB-4 score and chronic kidney disease in metabolically healthy men
Source: Sci Rep. 2022 Oct 5;12:16638. doi: 10.1038/s41598-022-21039-0 (PMC9535017; doi:10.1038/s41598-022-21039-0)
Supplement: Supplementary file 1 — Supplementary Information. [file 41598_2022_21039_MOESM1_ESM.pdf]

Potential link between high FIB-4 score and chronic kidney disease in a metabolically healthy men

Supplementary information

Supplementary Figure S1. Standardized mean differences of co-variances adjusted by propensity score matching

Supplementary Figure S2. The association between FIB-4 score and the rate of change in eGFR

Supplementary Table S1. Baseline characteristics of participants without the exclusion criteria before propensity score matching

Supplementary Table S2. Linear regression analysis with the rate of change in estimated glomerular filtration rate (eGFR) as the dependent variable in participants with one or more risk factors

Supplementary Table S3. The risk of CKD incident 5-year later according to the number of metabolic factor

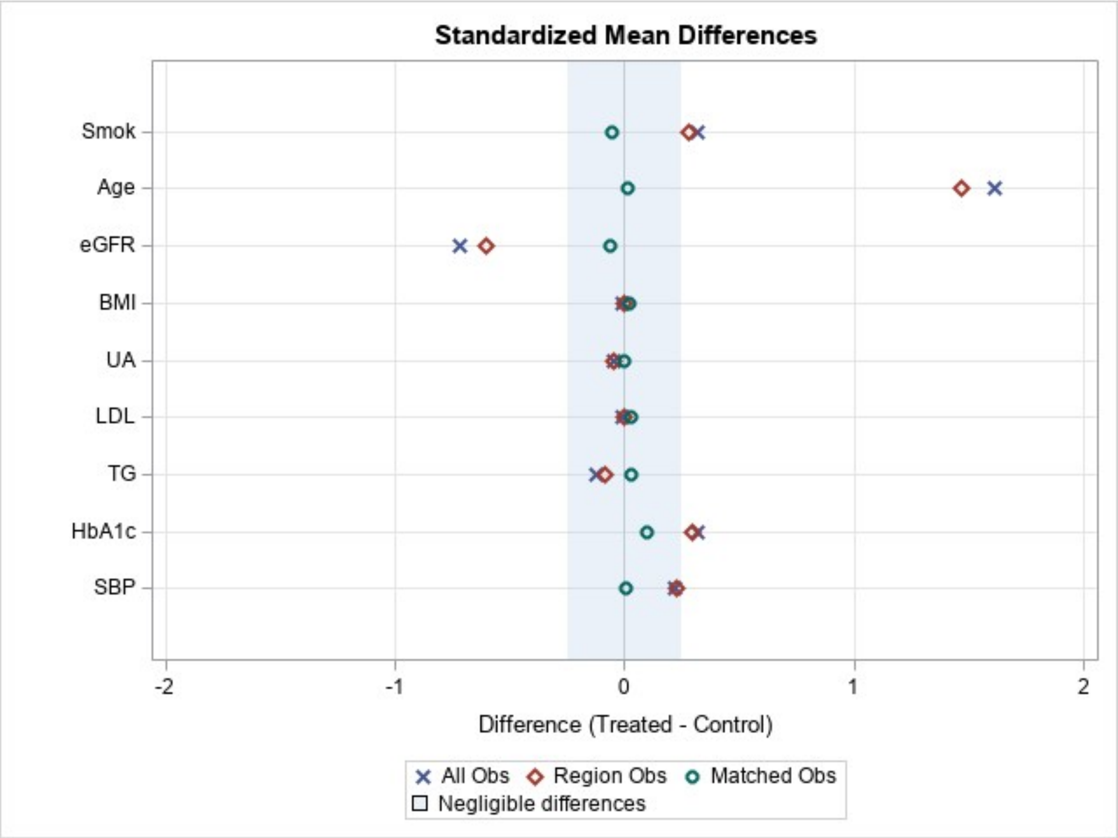

Supplementary Figure S1. Standardized mean differences of covariances adjusted by propensity score matching
The propensity score model includes the interaction of a habit of daily smoking (smok), age, basal estimated glomerular filtration rate (eGFR), body mass index (BMI), serum uric acid (UA), low-density lipoprotein cholesterol (LDL), triglycerides (TG), hemoglobin A1c (HbA1c), and systolic blood pressure (SBP). Standardized mean differences of matched participants are shown table 1.

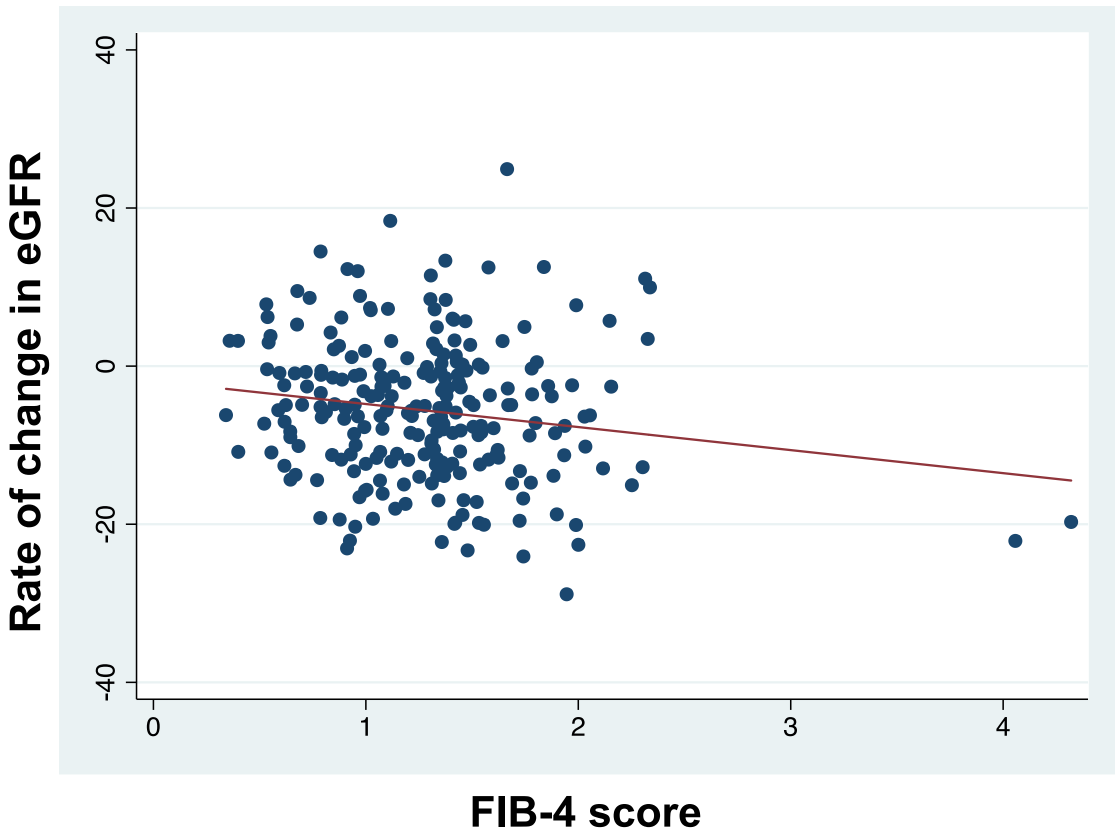

Supplementary Figure S2. The association between FIB-4 score and the rate of change in eGFR
The scatter plot for participants without specific metabolic abnormal (*N* = 237). The detail of linear regression was shown in Table 2. Coefficient = -2.9146 (95% confidence interval -5.1696 to -0.6596, *P* = 0.012). Rate of change in eGFR = ($eGFR_{2014}$ – $eGFR_{2009}$) / $eGFR_{2009}$. FIB-4 score was value at baseline.

Supplementary Table S1. Baseline characteristics of participants without exclusion criteria before propensity score matching

Participants, *n*	5,353
Age, y	38 (10)
Male, n (%)	5,353 (100)
FIB-4 score	0.73 (0.32)
Body mass index, kg/m2	22.9 (3.3)
eGFR, mg/min/1.73m^2	81.8 (12.5)
AST, IU/L	24 (12)
ALT, IU/L	28 (21)
γ-GTP, IU/L	35 (31)
Triglycerides, mg/dL	109 (77)
LDL-cholesterol, mg/dL	119 (32)
Platelet, x10^3/μL	254 (51)
Uric acid, mg/dL	6.0 (1.1)
Hemoglobin A1c, %	4.8 (0.5)
Systolic blood pressure, mmHg	118 (14)
Diastolic blood pressure, mmHg	72 (11)
Smoking, n (%)	1,964 (37)
Medication	
Hypertension, n (%)	161 (3)
Diabetes mellitus, n (%)	93 (2)

Smoking: participants of a habit of daily smoking
Medication for hypertension: participants of the usage of anti-hypertension medicine
Medication for diabetes mellitus: participants of usage of anti-diabetes mellitus medicine including insulin
Data: mean (standard deviation)
eGFR: estimated glomerular filtration rate, LDL: low-density lipoprotein

Supplementary Table S2. Linear regression analysis with the rate of change in estimated glomerular filtration rate (eGFR) as the dependent variable in participants with one or more metabolic factors

Variables	Simple linear regression			
	**Coefficient**	**95% CI**	**t-value**	***P* value**
FIB-4 score	.9085	-1.0261 to 2.8430	.93	.356
Age	.1168	-.0514 to .2849	1.37	.173
Body mass index	.1080	-.1846 to .4006	.73	.468
Triglycerides	-.0014	-.0189 to .0161	-.16	.874
LDL-cholesterol	-.0021	-.0393 to .0351	-.11	.912
Uric acid	.2077	-.6734 to 1.0888	.46	.643
Hemoglobin A1c	.1381	-1.2853 to 1.5614	.19	.849
Systolic blood pressure	-.0463	-.1044 to .0118	-1.57	.118

N = 227
Rate of change in eGFR = ($eGFR_{2014}$ – $eGFR_{2009}$) / $eGFR_{2009}$.
Metabolic factor: body mass index <25.0 kg/m2, hypertension (defined as systolic blood pressure ≥140 mmHg, diastolic blood pressure ≥90mmHg, and/or the usage of anti-hypertension medicine), or a habit of daily smoking.
eGFR: estimated glomerular filtration rate
LDL: low-density lipoprotein

Supplementary Table S3. The risk of CKD incident 5-year later according to the number of metabolic factor

	Number	Odds ratio	95% CI	*P* value
**Metabolic factor = 1**	**169**	**1.62**	**0.68 – 3.88**	**0.271**
BMI (-), HT (-), smoking (+)	72	0.94	0.18 – 5.01	0.943
BMI (-), HT (+), smoking (-)	58	2.11	0.61 – 7.38	0.240
BMI (+), HT (-), smoking (-)	39	3.14	0.46 – 21.57	0.245
**Metabolic factor = 2**	**46**	**0.41**	**0.11 - 1.52**	**0.180**
BMI (-), HT (+), smoking (+)	12	N/A	N/A	N/A
BMI (+), HT (-), smoking (+)	10	1.00	0.05 – 22.18	1.000
BMI (+), HT (+), smoking (-)	24	0.19	0.03 – 1.10	0.063
**Metabolic factor = 3**	**12**	**0.14**	**0.01 – 2.52**	**0.184**

Odds ratios were calculated after propensity score-matching. Metabolic factors included body mass index (BMI) ≥25.0 kg/m^2, hypertension (HT, defined as systolic blood pressure ≥140 mmHg, diastolic blood pressure ≥90 mmHg, and/or the use of antihypertensive medication), or daily smoking habits. CI; confidence interval, N/A; not applicable for statistical analysis because one of 12 participants had CKD incident.
